# Animal research nexus: a new approach to the connections between science, health and animal welfare

**DOI:** 10.1136/medhum-2019-011778

**Published:** 2020-02-19

**Authors:** Gail Davies, Richard Gorman, Beth Greenhough, Pru Hobson-West, Robert G W Kirk, Reuben Message, Dmitriy Myelnikov, Alexandra Palmer, Emma Roe, Vanessa Ashall, Bentley Crudgington, Renelle McGlacken, Sara Peres, Tess Skidmore

**Affiliations:** 1 Department of Geography, University of Exeter, Exeter, UK; 2 Department of Geography, Universities of Exeter, Exeter, UK; 3 School of Geography and the Environment, University of Oxford, Oxford, UK; 4 School of Sociology and Social Policy, University of Nottingham, Nottingham, UK; 5 Centre for the History of Science Technology and Medicine, University of Manchester, Manchester, UK; 6 School of Geography and the Environments, University of Oxford, Oxford, UK; 7 School of Geography and Environmental Science, University of Southampton, Southampton, UK; 8 Science and Technology Studies Unit (SATSU), Department of Sociology, University of York, York, UK

**Keywords:** medical humanities, cultural history, sociology, veterinarian, medical ethics/bioethics

## Abstract

Animals used in biological research and testing have become integrated into the trajectories of modern biomedicine, generating increased expectations for and connections between human and animal health. Animal research also remains controversial and its acceptability is contingent on a complex network of relations and assurances across science and society, which are both formally constituted through law and informal or assumed. In this paper, we propose these entanglements can be studied through an approach that understands animal research as a nexus spanning the domains of science, health and animal welfare. We introduce this argument through, first, outlining some key challenges in UK debates around animal research, and second, reviewing the way nexus concepts have been used to connect issues in environmental research. Third, we explore how existing social sciences and humanities scholarship on animal research tends to focus on different aspects of the connections between scientific research, human health and animal welfare, which we suggest can be combined in a nexus approach. In the fourth section, we introduce our collaborative research on the animal research nexus, indicating how this approach can be used to study the history, governance and changing sensibilities around UK laboratory animal research. We suggest the attention to complex connections in nexus approaches can be enriched through conversations with the social sciences and medical humanities in ways that deepen appreciation of the importance of path-dependency and contingency, inclusion and exclusion in governance and the affective dimension to research. In conclusion, we reflect on the value of nexus thinking for developing research that is interdisciplinary, interactive and reflexive in understanding how accounts of the histories and current relations of animal research have significant implications for how scientific practices, policy debates and broad social contracts around animal research are being remade today.

## Introduction

This paper explores new ways of making connections around a controversial topic of research in the social sciences and humanities that is characterised by both complex links and stark divisions. The Animal Research Nexus Programme was funded by the Wellcome Trust (2017–2022, grant no: 205393) to support collaborative work investigating the historical dimensions and social relations of animal research in the UK. It aims to deliver new research and public engagement exploring the changing ways in which scientific practices, research governance and public imaginations connect the, often divergent, domains of science, health and animal welfare. Pursuing this through a collaborative programme of work means we are also experimenting with interdisciplinary, interactive and reflexive ways of working across science, social science, the humanities and policy. In this topic, and in our approach, we hope to contribute to the Wellcome Trust’s strategic aim to ‘bring new perspectives and ways of thinking to the historical, ethical and cultural contexts in which medical science takes place’.1 We first outline why a new approach to the social and historical relations around animal research is needed, before expanding on our development and use of the concept of the animal research nexus as an integrative approach in the rest of the paper.

Animal research in the UK is often characterised by highly polarised debates, structured around strongly proanimal and antianimal research positions. This polarisation captures an important public dimension to the history of British animal research. Yet, this narrative also obscures the deep historical entanglements between people and positions through which animal protection groups, scientists and policy-makers created the frameworks currently regulating UK animal research.2 Drawing these connections is further complicated by a division in the animal studies literature between normative animal ethics and empirical studies of the changing ways in which care and ethics are enacted in practice. For example, critiques of the utilitarian frameworks used to authorise animal research, like harm-benefit analysis,3 can mask the complex ways in which ethical responsibilities are distributed and enacted around structured decision-making processes.4 And, while earlier laboratory ethnographies identified the social divisions of labour separating practices of animal research and care,5 more recent work reveals how increasing demands for openness and translational research extend responsibilities for care across professional roles.6 These disjunctures mean it is difficult to tell a story about the development of animal research in the UK that is historically accurate, socially and ethically nuanced and widely legible. Yet, the stakes of such narratives are high. How the histories and current relations of animal research are recounted in public and academic research has significant implications for how scientific practices, policy debates and the broad social contracts around animal research are made and remade today.

In this paper, we explore the challenge of developing a conceptualisation of animal research that is able to encapsulate the connections and disconnections in this field, while also seeking to sustain a productive dialogue between the social sciences and humanities and those involved in animal research and regulation. To do this, we propose the concept of the animal research nexus. For us, the animal research nexus refers to the inter-relations between scientific research, human health and animal welfare, held together through ethical practices and social norms embodied in governance, regulation and care. This definition of the animal research nexus indicates the importance of both interdisciplinary and interactive research: the former develops understanding of how connections are made between the domains of research, health and welfare, while the latter allows for consideration of how governance is informed by wider social contracts. The combination of the two necessitates an ongoing and reflexive discussion about gaps between regulated practices and societal expectations of governance. In outlining an approach to study the animal research nexus that is interdisciplinary, interactive and reflexive in this paper, we seek to understand the connections, disconnections and potential for change in the way animal research is practised by scientists, managed by policy and imagined by publics.

The social sciences and humanities have an important role to play in understanding the multiple dimensions to animal research; however, the challenges presented by the study of animal research are not unique. There are many other controversies in the contemporary medical humanities where there is a need to both encapsulate complexity and enable dialogue that is inclusive of different scientific, policy and public dimensions.7 This search for ways out of disciplinary silos has resulted in a proliferation of vocabularies around interdisciplinarity; yet many of these erect further barriers to communication with policy and the public. By contrast, the concept of nexus is increasingly used in other areas of interdisciplinary environmental research, in part because of its accessibility to policy-makers. Yet, there is also a need to attend carefully to what Barry *et al* call the ‘logics of interdisciplinarity’ that are enacted in the nexus concept.8 We propose that the interdisciplinary, interactive and reflexive logics in the animal research nexus can function as a site for rethinking the practices of collaborative research. We further suggest that our use of nexus thinking is not determined by policy priorities, but nonetheless remains attuned to and in conversation with them and is committed to producing research that can actively contribute to the development of policy agendas. Nexus thinking, following understanding of interdisciplinary research by Barry *et al*, might be closely associated with the ‘logics of innovation and accountability’, but importantly ‘it is not reducible to them’.9

In what follows, we elaborate our argument about the animal research nexus by drawing first on the experiments with nexus thinking that have characterised the field of environmental research. There are opportunities to learn from the proliferation of nexus approaches applied to food, water, security and more, as a way of understanding the complexities of interdisciplinary research and the challenges of governance.

## Nexus approaches within and beyond environmental research

Nexus approaches have been developed in environmental research, for example, around the emblematic water-energy-food nexus, as a way of understanding how multidimensional systems are constituted by interdependent relations that frequently involve conflicting values.10 Complexity and systems science have significantly informed how nexus approaches are positioned as the antithesis to disciplinary silos through fostering opportunities for more collaborative research and policy to address multidimensional challenges.11 Nexus thinking has been used to support the ‘holistic treatment of interdependent sectors or systems’12 and encourage recognition that ‘transformations or developments in one sector, inevitably create reverberating repercussions, be they adverse or favourable, in other sectors’.13 Nexus approaches have also been used to suggest that transformative ways of working are required to reconnect disciplines, build capacities within and outside of science and effect change across governance and culture.14 Used in this way, such approaches can open up discussion of the material and political interdependencies, tensions and trade-offs that need to be addressed when dealing with complex interconnected environmental systems.

In the environmental sciences, nexus approaches have been most fully developed in studying the inter-relations of water, energy and food systems. This research shows how understanding complex and changing human-environmental systems, like agriculture, requires cross-disciplinary work which can raise challenges for management practices, like cost-benefit analysis, that are usually focused on individual natural resources. The concept of nexus is used to refer simultaneously to a set of environmental inter-relations, an interdisciplinary research methodology and an integrated approach to policy.15 However, this work also demonstrates that the logics of interdisciplinarity, interactivity and reflexivity do not always proceed hand in hand.16 Nexus approaches in environmental research are thus instructive for understanding how to develop approaches that both deepen interdisciplinary practices *and* retain critical space for discussion of conflicting values.

Applying an approach that takes interconnections as its starting point does raise questions, which are also seen in other relational approaches like actor-network theory, around where analysis should start and end. Stein *et al* 17 suggest that acknowledging that different systems and domains are interconnected can quickly end up with analysis connecting everything to everything else. In reality, the main issue with nexus approaches to date is that they have been used to close down discussion too quickly. For example, environmental nexus studies have prioritised natural scientists to speak for the materials that constitute the nexus, rather than opening up their sociopolitical implications. This has led some to suggest nexus work has developed along ‘technocratic and reductionist’ paths,18 focusing on the intersections of material systems, overlooking political contestation and producing analysis devoid of the people affected.19

A sole focus on interdisciplinarity is insufficient to progress a nexus approach. Interactivity is equally needed to understand the people, practices and policies that are implicated in the constitutive domains of a particular nexus. Reflexivity, too, is required to understand how well research methods study, and management approaches manage, the critical connections therein. This requires wider engagement with the political, economic and cultural framing of decisions and involvement with those affected,20 incorporating their values, beliefs and experiences. The critique that nexus is simply a buzzword, adding little analytical power to existing languages of complex systems and interdisciplinarity,21 can be upheld if nexus approaches are applied without also working in interactive and reflexive ways with those affected by them. We propose below that there is particular value in extending this relational requirement to consideration of health and the contribution of the medical humanities.

Human and animal health and well-being are already central to much nexus thinking in the environmental sciences, including explicitly, such as in the ‘agriculture-nutrition-health nexus’.22 Some of this work takes inspiration from the water-energy-food nexus and incorporates greater attention to the material practices and social values associated with agricultural livelihoods, food cultures and human and environmental health. Others use the vocabulary of nexus to further so-called ‘one health’ approaches, which recognise the social and material connections between humans, non-human animals and environmental health.23 There is a further resemblance to other relational approaches to human and non-human animal (hereafter, animal) health, such as those informed by actor-network theories (ANT), assemblage-style rhizomatic approaches24 or posthumanist perspectives.25 All the above lend themselves to strengthening interdisciplinary approaches for understanding the affective and material relations between multiple species that contribute to health. However, while all of these conceptual approaches have deepened insights into the links between human and non-human animal health, not all terms (such as assemblage) move easily into conversations with policy or publics.

Our use of nexus is thus not a rejection of conceptual relational scholarship around multispecies health, but rather an attempt, following Williams *et al*, to mobilise ‘the conceptual and methodological insights of assemblage thinking to advance research on nexus issues’26 and engage this diverse intellectual lineage within interdisciplinary-focused, policy-focused and public-focused research. The idea of the animal research nexus provides a way to convey the complex, contradictory and contested interconnections between scientific practices, human health and animal welfare into conversations between disciplines, policy and the public. We propose the nexus itself as a starting point, both to recognise the importance of these interconnections, but also to avoid prioritising the status of either biomedical research or animal welfare in advance and avoid recapitulating the polarisations around animal research. There is an invitation for different disciplines to think about how understandings of human health are entangled with animal welfare science and the practices of animal care, how knowledge about animals also develops through biomedical research and how both are complexly enmeshed in the changing cultures, economy and governance of scientific research. As Stein *et al* argue, ‘what is new about the nexus approach is that it considers multiple sectors equally important […] providing multiple entry points for actors from different policy sectors to get involved’.27 We suggest the mobility of the concept of nexus, across disciplines and into policy, ‘has the power to open up new spaces for critical debate’28 and draw together previously disparate actors in addressing broad, complex and entangled systems.

We also propose the humanities have an important role to play in opening up nexus approaches and attending to how the values and experiences of those affected by these issues are included and interpreted. Earlier nexus approaches have been criticised for methods that prioritise ‘quantifying and cataloguing the relationships between constituent elements’,29 which the humanities can supplement through an openness to different voices, affects and experiences. The humanities also have a vital contribution to make in understanding the centrality of dialogue and debate, and thus reflexivity, as an integrative imaginary within nexus thinking. The discussion of nexus within the context of cultural poetics by DuPlessis and Quartermain’s30 is valuable here. In their conceptualisation, nexus thinking ‘allows one to appreciate difference and disparity, to pinpoint perhaps radical disagreements, to attend to rupture as well as continuity and to dispersion as well as origin’.31 The inclusion of the humanities in nexus approaches is thus vital for thinking about how dialogues open to difference can be staged, so retaining an interpretative openness. In this view, nexus approaches provide a mode of working, ‘a shifting place of dialogue, debate and reconfiguration’,32 that enlivens the study of interconnectivity in ways that can address unstable, difficult, technical and governmental complexities.33 Rather than privileging the sciences in setting the parameters for how integration is to be understood, DuPlessis and Quartermain draw on the humanities to conclude that a nexus is ‘a continuous and continuing construction that embraces contradiction, variousness and dispute’.34 They argue that inter-relatedness can be teased out in ways that support interdisciplinary dialogue and new ways of thinking and problem-solving.

The potential for nexus approaches to encapsulate both integration and contradiction fits well with our commitment to researching the close connections and ongoing disputes around animal research. In the ‘Nexus thinking in previous work on animal research’ section, we develop this argument through introducing some precursors to a nexus approach in the study of animal research.

## Nexus thinking in previous work on animal research

The humanities and social sciences have long looked to animal research as a key site for understanding how new kinds of animals, novel ways of knowing humans and innovative ways of managing bodies are forged through scientific and social practices. Over 30 years since Lynch’s classic study35 of the conversion of the animal body into a scientific object, there are now multiple accounts of the complex transformations occurring in and around the animal research laboratory.36 These vary widely in their aims and objects of study, encompassing social histories of scientific institutions,37 sociological accounts of experimental practices,38 ethnographies of animal care,39, 40 philosophical work on model organsims and critical challenges to research governance and the calculation of ethics.41 This diversity indicates the material importance and imaginative pull of the animal research laboratory as a space for studying the remaking of human-animal relations and ethical practices in an era of modern biomedical science.

There are previous applications of the language of nexus to animal research in the existing literature, which use ‘nexus’ informally to indicate these connections and links. These previous applications are useful for indicating the potential of combining connections in a new conceptual approach, and fall broadly into three distinct categories. The first emphasises the social and material connections between humans and animal worlds. Applied informally, the term ‘nexus’ appears as a useful synonym for approaches that recognise the entanglement or assemblage of material and affective relations that shape human and animal encounters, as in Wilkie’s piece on the human-animal nexus, and in ‘one health’ approaches (see ‘Nexus approaches within and beyond environmental research’ section).42 More narrowly, the language of nexus has been used to consider how health across species is made and unmade together through the associations found between domestic violence and animal abuse.43 This use of ‘nexus’ mirrors moves in environmental research by bringing together domains of enquiry formerly considered separately, in this case human, animal and environmental health. However, applied to animal research in this way, the language of nexus can reproduce the same problems of reductionism encountered in environmental studies, with critical commentary emerging around the restricted ideas of integration found in one health, one medicine or one welfare approaches.44

The second informal use of ‘nexus’ in literatures around animal research are found in calls for more interdisciplinary research. These are primarily authored by scientific practitioners seeking new ways of working together within the sciences or across the sciences, humanities and social sciences. For example, Blake *et al* suggest the Mouse Genome Database can act as an integration ‘nexus’ for the different research communities using mice as a model organism to address complex relations across contemporary biological research.45 Lund *et al* make a broader call to work across a ‘nexus’ of the social and natural sciences in order to develop new opportunities in laboratory animal use and welfare.46 These calls indicate both a history of separation and a new move from the research community to forge platforms for interdisciplinary research. These may call for involvement of the humanities and social science research. However, they rarely incorporate the social, ethical or political questions raised by interdisciplinary work directly.

The third implicit use of a nexus vocabulary is more directly political. This is often found in the social sciences and humanities and posits a ‘nexus’, or connection, between the production of animal and human subjects through the practices of animal research, making explicit links between animal research subjects and human political subjectivities. The ‘nexus’ here is generally a social and political one, in which animal research is used as a case study to understand the entanglement of specific identities and the alignments of interest in different political situations. This sort of ‘nexus’ has been used to understand the wider historical conditions that underpin a particular controversy, as in discussion of Lansbury’s classic work ‘on the symbolic political nexus between animals, feminist suffragettes and labourers that was central to the antivivisection riots’ by Jamison and Lunch in 19th century London.47 More narrowly, the idea of ‘nexus’ has been applied to identify how different voices are included and excluded in the organisation of animal research, which acts to exclude certain forms of identity and argument from debates. As Michael and Birke suggested in 1994, there are ‘discourses of exclusion and inclusion which form a flexible nexus through which the core set can be demarcated’.48 This focus on the political, economic and cultural nexus of animal research both situates past controversies and helps understand the continuation of contemporary divisions.

Although informally used in all three instances, the use of the term nexus in past work on animal research has important parallels to the nexus thinking in environmental research introduced above. In summary, nexus is used to refer to the interconnections between previously distinct domains of enquiry, the value of interdisciplinary approaches and the importance of attending to how power shapes both connections and exclusions around animal research. This diversity of use may reinforce the views that nexus is an imprecise and unhelpful term.49 However, our reading is that the diversity of nexus thinking around animal research is not indiscriminate. Rather, it connects the different relationalities of animal research, which are material, disciplinary and political. We suggest that there is significant potential in explicitly considering these relationalities together.

At present, there is a rich body of work in the social science and humanities on laboratory animal research. However, they are frequently disconnected from each other empirically, analytically and also from policy. There is also no clear framework for comparative consideration or articulation with the wider social, economic, political and institutional practices of science.50 Case studies are often embedded in different national political contexts, with analysis distributed across topics in the social sciences and humanities. This means it can be difficult to draw comparisons and policy lessons across them. To give one example, Haraway’s reformulations of ethics, which draws on American case studies to foreground individual responsibility and agency in contexts where animal use is regulated internally within research institutions,51 cannot travel without modification into European landscapes dominated by centralised legislative and licensing processes.52 In identifying the potential for applying nexus thinking to animal research, we want to explore the opportunity for developing a vocabulary and approach that can bring together the different relational elements of animal research in and across particular places. Our own collaborative work focuses on the development and implementation of the UK Animals (in Scientific Procedures) Act in 1986 (hereafter, ASPA), but we anticipate the approach we develop below can be used to understand the history and current practices of animal research elsewhere.

## Developing a nexus approach to laboratory animal research

We suggest that a new approach to the challenges facing animal research is timely in light of the growing demands made of biomedical research around trust, openness, dialogue, care, responsibility and access to health. These issues feature in many social and technological controversies studied by the medical humanities, but they have a specific configuration in UK animal research. Public conversations in the UK around animal research are starting to change, as research is now expected to be more open,53 more engaged54 and in many cases more translational.55 This increased openness has a distinctive trajectory given local histories of secrecy around animal research, ongoing public mistrust and past polarisations that have impeded engagement across different positions and perspectives. There are also societal challenges emerging across the domains of science, health and animal welfare. Questions exist in relation to animal welfare, as the continuing abundance of genetically altered animals confounds social expectations of commitments to replace and reduce the use of animals in research. An emergent ‘replication crisis’, alongside increased concern about ‘questionable research practices’,56 is confronting professionals and policy-makers charged with assuring the quality and social responsibility of biomedical science.57 The UK postwar social contracts around access to health benefits are also changing, as biomedical research becomes progressively personalised and healthcare increasingly politicised.

At the same time, a key starting point for our work with nexus approaches is that animal research in the UK is *already* situated and governed through policies that seek to connect practices across the domains of science, health and animal welfare. For example, ASPA is implemented through regulatory techniques that strive to join up policy and practice across scientific research, animal welfare and research benefits, such as human and animal health. The licensing of animal research under ASPA requires researchers to consider their ability to replace, reduce and refine (the 3Rs) in the use of animals in their work. This in itself connects science and animal welfare, while integrating the potential horizon of replacement for both science and society. UK government regulators review each application using a harm-benefit analysis, which connects the harms and benefits of research, while also opening up questions around the changing social acceptability of both harms and different research benefits like human, animal and environmental health. Finally, there is a growing focus in regulation on fostering a culture of care, in both research and clinical contexts, recognising that while the regulatory 3Rs guidelines for ‘good’ humane science are important, they cannot fully account for the performance of care practices.

Understanding the complex dynamics of public and policy engagements with animal research requires a new approach, which the Animal Research Nexus Programme aims to create. The Animal Research Nexus Programme consists of 15 people based across 5 UK institutions. While we all have disciplinary training, we each share a commitment to interdisciplinarity that predates our collaboration on this Award. Several of us are, or have been based, within science faculties and most of us have worked closely with natural or clinical scientists throughout our careers. This experience is vital to our engagement with the concept of nexus and our ability to look across the multiple facets of the animal research debate. We also share a commitment to interactive research in this area. For example, several of the authors were previously involved in developing a collaborative agenda58 for future research using the methods of the humanities and social sciences to research laboratory animal use and welfare. This interactive priority-setting process confirmed that the use of animals in research is already subject to ongoing questioning by researchers, policy-makers and animal protection groups. Finally, we consider that a nexus approach to animal research needs to be reflexive. Social and historical inquiry on animal research is increasingly an integral and interactive part of this complex field. This is not new; the original formulation of the 3Rs emerged from interdisciplinary research in the 1950s, reaching across the ‘two cultures’ of the sciences and humanities.59 However, the use of social science enquiry and marketing research is increasingly informing the multiple dimensions of animal research today,60 from the use of polling and survey data, to engaged qualitative research.

**Figure 1:**
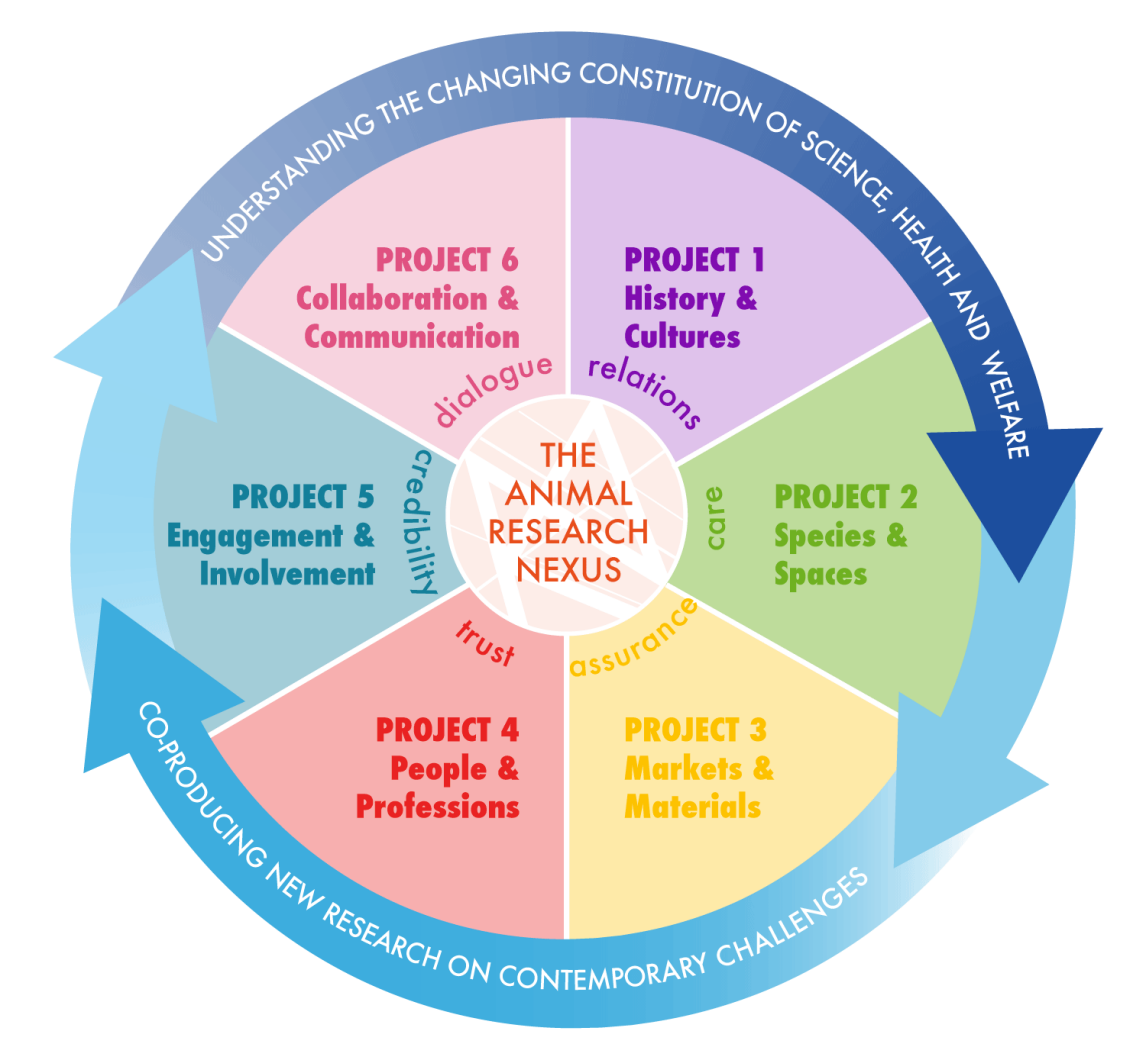
The structure of the Animal Research Nexus Programme.

We have taken the multiple dimension of a nexus approach and translated them into a programme of work, which consists of interlinked projects and new empirical research (Figure 1). Project 1 explores the historical evolution of the regulation around UK animal research. Projects 2 and 3 consider how the inclusion of novel species, field spaces and biotechnological innovations in the supply of research animals present challenges to care that have to be managed in the day-to-day practices of animal research. Projects 4 and 5 trace the changing ways in which professional and public representatives are authorised and trusted to speak for issues around the nexus of science, health and animal welfare. These explore the role of the Named Veterinary Surgeon (NVS) and the growth of patient and public involvement in research in turn. Connecting projects and engaging a wide range of project partners, stakeholders and publics, we have a further strand of work on communication and collaboration, which devises novel public engagement methods to resist the pressure to resolve dispute by removing difference, instead seeking to involve different voices and evolve the terms of the debate.

Overall, we suggest that the attention to complexity in the nexus approaches used in the environmental sciences can be further enriched through conversations with the social sciences and medical humanities. In what follows, we explore three different analytical cuts through the animal research nexus, thereby demonstrating first the value of this concept for charting and writing the history of animal research, second for considering changing patterns of connection and inclusion imagined and enacted through governance and finally for introducing the shifting affective relations that are being enacted to accommodate new demands for openness and care.

### Understanding continuity and change over time

Given the complex relations we explore are dynamic in time as well as space, historical analysis needs to be integral to the nexus thinking. Historians deal with continuity and change and highlight the contingency of the current state of affairs—things could have plausibly developed differently. Many arrangements now integral to animal research in the UK are recent developments. Some were explicit changes in regulation—most notably ASPA 1986 that replaced the previous 1876 Cruelty to Animals Act, and its multiple updates since 1986, including alignments with EU regulation. Other changes were subtler. For example, the current integral role of the NVS reverses the initial distancing of the veterinary profession in the debates leading to the Victorian legislation, and it was not entirely envisioned in ASPA—other ‘suitably qualified’ experts could have been responsible for animal care. Likewise the 3Rs is now a prominent principle in ASPA and its guidance documents, but was not explicitly invoked in discussions leading to ASPA’s development. The specific regulatory nexus of relations between science, regulation and animal welfare will have evolved differently elsewhere.

As well as change and contingency, continuity within the animal research nexus cannot be taken for granted but invites explanation. While the intensity of the animal experiment debate has waxed and waned, the tensions between scientists, animal welfare advocates and antivivisectionists have persisted since at least the 19th century. Moreover, animal research itself has a strong cumulative dimension, or path dependency, which can be captured by a nexus approach that encompasses the importance of materials, information and norms built over time and shaping practices in the present. Knowledge made with a handful of ‘model organisms’ has accumulated,61 alongside associated nomenclatures, infrastructures, husbandry techniques, communication strategies and practices of care,40 making specific species attractive to new generations of scientists. The importance of this continuity is highlighted by the work and investment required when new species are brought into laboratory research. Furthermore, this cumulative character of animal research poses a challenge to developing alternatives, as non-animal approaches have to acquire scientific credibility, and work against major investments in animal models. Seeing these patterns in a longer historical perspective allows for more meaningful interpretation and intervention.

A committed historical sensibility is therefore crucial in making sense of the relations, tensions and controversies in the animal research nexus, recognising that these are not given but change in time and are channelled by past relations. But what kinds of animal-oriented histories can feature within a nexus approach? The emphasis on the tangled social, ethical, political and legal alignments around laboratory animals has already attracted much useful historical analysis. Historians of biology and medicine have emphasised the relations between experimental animals and scientists to a great degree, highlighting the epistemic and cultural roles of animals in the scientific enterprise, as well as the importance of infrastructures, laboratory environments and communication.62 Histories of laboratory animal technicians, veterinarians and welfare practices are fewer, but have highlighted the many people beyond research scientists who play crucial and changing roles.63 This literature informs our study of the nexus, but paying attention to multiple perspectives and inter-relations can bring these accounts together. The aim is not to be exhaustive or abandon microhistory; rather, the nexus approach can highlight the diversity of actors and tell new stories with broader sets of agents, underwritten by collaborative work. The focus on the entangled legal processes, lobbying, public controversies and laboratory practices involved in the shaping of ASPA, and its effect on laboratory animal science in the UK, is one such story.

Second, nexus thinking can contribute to the exciting but conceptually challenging historical work on bonds between humans and other animals, often framed as animal agency. Some have argued the task of reconstructing animal subjectivities is impossible64; others have focused on resistance65; others still turn to the natural sciences, especially ethology,66 raising significant epistemological questions for those fluent in the history of science. Appeals to the natural sciences sit uncomfortably with the knowledge that science and medicine, like all other areas of human culture, are themselves products of history. They also require a potentially valid but ill-justified assumption that animals—especially those in close contact with humans—have retained an immutable essence throughout the past. More recent historiographic interventions call for a distinct approach to reconstructing animal agency, embedded in humanities and social sciences, inspired by ANT and animal histories,31 35 36 and going beyond thinking of animals as just resisting. All these interventions emphasise the view of animal agency as relational—what is to be explained is not so much what animals do, but how the inter-relations between humans and non-humans generate change or maintain continuity. This perspective, inherent in the nexus approach, can also highlight the diversity of human-animal relations. For example, while the language of labour is often used to describe human relationships with certain animals, such as service dogs, in the laboratory this language might deflect from a distinct social and interspecies arrangement in which animals are subject to care, but also killing, within elaborate regulatory, ethical and affective frameworks. Focus on interspecies relations within the nexus should generate specific, historically situated accounts, but in ways that can facilitate reading across contexts.

Finally, the benefits of interdisciplinary research involving close collaboration between the humanities and social sciences should not be underestimated. For example, close links with ethnographic work can highlight voices that are not easily accessible or present in archival records—those of animal technicians, activists or officials involved in regulation and laboratory compliance. Ethnographies can bring to the fore the issues of spatial arrangement, affective relations and moral identity-making, whose genealogies can be traced through reading historical sources in a new light. Appreciating current debates and anxieties—or lack thereof—can highlight change and reveal problems in need of explanation. Such appreciation, moreover, can help historians offer more effective contributions to current debates, without abandoning the crucial commitment to treat the past on its own terms. What is offered to a historian is thus a broader understanding of the present, and new questions. For example, how have histories of animal research worked to shape and reshape the present as a rhetorical device? Was ASPA conceived as a ‘mouse act’? If the 3Rs did not feature much in the 1980s debates, was the thinking behind them influential but left implicit in pursuit of compromise?

These are historical questions, but they have political consequence for the way history is mobilised today to frame present practice and shape future policy. Nexus is an invitation to mobilise history to serve the present and shape the future in an explicit, guided and collaborative sense. It encourages working within the object of study as opposed to framing historical methods pseudo-scientifically as being in some way objectively distinct. Far from weakening the rigour of historical method, nexus approaches can strengthen history as a discipline while allowing critical contributions with the potential to effect change in the present, and in doing so shape the future. History and the medical humanities help remind us that the connections and disconnections we seek to chart through a nexus approach have both path dependencies and local specificities that require interdisciplinary work to fully understand.

### Charting inclusions and exclusions in governance

The previous section showed how a nexus approach involving history can help highlight and reveal questions of continuity and change through interdisciplinary work. In this section, we explore how nexus thinking can also answer political questions around the ways different voices and positions are included or excluded from the governance of animal research. We consider how animal research incorporates values, beliefs, experiences and power relations through both formal and informal mechanisms which shape changing ideas as to whom and what should not be included. This section thus indicates how a language of nexus can contribute to attempts to be more attentive to patterns of inclusion and exclusion in work on entanglement.67 It can also be used to situate the UK animal research nexus within a complex and evolving political context, reflecting on how far UK animal research regulation should be used internationally as a model of good governance, as some have suggested.68

A conventional policy network analysis of UK animal research governance would identify what Lyons calls a ‘policy community’, which is insulated from interests including Parliament, the public, animal protection groups and animals themselves.69 This approach takes into account how ‘macro-level factors such as the broader power distribution in society or national political institutions set the context that constrains and facilitates certain forms of network’.70 Like a nexus approach, this analytical lens requires careful attention to history. However, unlike nexus thinking, certain inputs are understood as causal, rather than broadly co-constitutive of policy outcomes. Other authors have also studied the politics of interests: Raman *et al* 71 explore how social movements are sometimes excluded from scientific policy processes as they are deemed to represent the ‘minority’ interest, rather than being viewed as advocates for a different kind of science that may be in the wider public interest.

Such interest-based approaches have much to offer our understanding of policy processes. However, here we consider how they might be seen to feed into a broader nexus type thinking. This thinking would demand close attention to the inter-relationships and dependencies between science, health and welfare, including the way in which stakeholders and publics are imagined through regulatory practices, and the historic way in which these connections have been forged. Here, we consider this approach via three specific examples.

First, how does legislation draw boundaries between what types of bodies are included and excluded from categories of regulatory protection? This differs across national contexts, often for reasons to do with the operation of governance, rather than a priori ethical commitments. In the UK under ASPA, experiments which use certain species (primates, horses, dogs, cats) require further justification. A nexus approach encourages us to look at the complex, contingent nature of this provision. Many of these animals can arguably experience greater suffering, according to scientific understandings of sentience and assumptions about welfare. However, this legislation also responds to the imaginary of public concern, previously defined as ‘societal sentience’,72 where society itself is assumed to experience varying levels of ethical harm. One could dismiss this special protection as speciesism, as have many critics.73 But our point here is that nexus thinking encourages us to consider how things could have been different or could be in the future, for example, as ideas about sentience or publics are transformed over time.

Second, a nexus approach enables a focus on how certain ways of knowing are included or excluded from policy-making and delivery. According to ASPA, all regulated procedures must be assessed to see whether ‘the harm that would be caused to protected animals in terms of suffering, pain and distress is justified by the expected outcome, taking into account ethical considerations and the expected benefit to human beings, animals or the environment’.74 An ethical analysis of this policy could suggest that animal research regulation conforms to a narrow utilitarian calculus.75 However, this critique assumes a particular understanding of what ethics is or where ethics is located, ignoring how harm-benefit analysis ‘operates within a wider set of interlocking social, ethical and regulatory frameworks’76 that recognises some intrinsic animal rights, considers the importance of a culture of care and might be expanded to engage a range of wider societal concerns. A nexus approach thus takes a broader view of how further ‘ethical considerations’ have accrued around regulation in one context, and not others, while considering the historical, scientific and societal drivers of what this means. It also encourages a focus not just on entanglements, but how ‘when one apparatus instantiates a particular world another is necessarily excluded’.77 For our work, then, nexus thinking affords the opportunity to explore what connects and flows between the spaces of governance, including but not limited to ethics committees, and how this relates to the lived, embodied experience of those with regulatory responsibilities. For example, current work is exploring how NVS manage the issues of identity created by their multiple responsibilities in relation to animals under their care.78

Third, our work is attuned to the ways in which certain voices are included and excluded from animal research governance. In the UK, public participation is largely enacted through bi-annual national opinion polls which are often cited by stakeholders, such that the polls have become a dominant technology of legitimacy.79 However, as touched on earlier, this figure of the ‘public’ is constructed through contrast with certain groups, such as social movements, revealing a public imaginary based in professional and political neutrality. Other routes are via lay participation in the local ethical committees known as animal welfare and ethical review boards, and the increasingly common patient representatives who may also be involved in reviewing health research that involves animals. This leads to difficult questions about representation, and whether lay members are expected to somehow give voice to animals, or to affected patients.80 Even if there was consensus on the latter, it remains unclear how societal or patient concerns would be defined or identified. Indeed, this challenge is recognised in a recent government report.81 This report committee was chaired by the first author of this paper, exemplifying how social scientists are now an integral part of the nexus. Those who take a nexus approach are perhaps particularly likely to be working closely with policy in their research and professional activities. It is therefore even more important to apply the same principles of contingency, coproduction and reflexivity to our own role in the nexus, as we argue for in relation to our research data.

In summary, nexus thinking encourages a broad analytical approach to policy and regulation, which goes beyond single disciplinary perspectives, for example, around political interests or ethical theory. Ultimately, this approach also requires us to adopt researcher reflexivity. The advantage of this type of approach is that it enables us to ask critical questions, for example, around whether the regulation of animal research adequately address social and scientific concerns and to identify gaps where new conversations may be needed.

### Distributing affects and changing political atmospheres

In the final section, we turn to questions around how attunement to the affective relations and political atmospheres82 of animal research are important analytical dimensions of a nexus approach. We consider the process of attunement that is crucial to developing a better understanding of how animal research structures feelings and generates meanings inside and outside research facilities. When dealing with animal research, it is impossible to avoid the negative affects (fear, pain, suffering, shame and stress) that circulate between humans, animals and environments, and how these have shaped the infrastructures, policies and communications around animal research. Equally, it is important to consider the role and distribution of positive affects (whether hope, curiosity, care or empathy) in motivating researchers, engaging patients and funding research. These affective dimensions are important for understanding how connections and disconnections are forged, and the role of emotions in shaping human-environment and human-animal relations more broadly.83 In what follows, we outline how recognition of these affects is central to the interdisciplinary study of animal research, reflect on how a nexus approach can help understand the distribution and institutionalisation of affects, and close with the importance of affect for shaping changes around the animal research nexus.

Scholars in the social sciences and humanities have increasingly looked at the operation of affects within ethnographic studies of animal research84 in order to trace the distribution of care and responsibility within research85 and understand what motivates a concern for and engagement with animal interest and agency.86 Kirk’s work on the history of animal stress demonstrates how stress facilitated the emergence of a ‘science’ of animal welfare through revealing the complex interdependencies between organisms and their physical and social environments in the 1950s.87 More recently, social scientists have been collaborating with animal welfare scientists on ways of assessing and measuring the affective states of the research animals to trace how research procedures, research funding decisions and unforeseen events affect their well-being.88 There is a growing focus on occupational stress, recognising the emotional labour demanded of animal technicians and others responsible for animal care and culling and placing this at the complex intersection of regulatory, professional and moral imperatives to care.89 There is also increasing interest in understanding the affective motivational states of scientists. This moves analysis beyond a traditional focus on institutionalised recognition and reward systems90 to incorporate the role of curiosity in driving research,91 even to characterise empirical science as what Daston calls a ‘web of affect-saturated values’.92 Finally, the growing involvement of patient representatives in decisions around research priorities adds further dimensions to the mobilisation of affects, whether calming fears or legitimising hope.93 A nexus approach offers the opportunity to frame these affective practices as a central organising principle of relations around animal research, rather than as a secondary effect.

A nexus approach allows us to expand the scale at which we study the affective landscapes of animal research to attend to how these affects are currently managed and distributed, and how they may be redistributed. These affects have become materialised through the built architectures, policy processes and even scientific practices of animal research in ways that make them differently visible across different public and private spaces. The evolution of the animal facility, separate from the research laboratories and obscured from public view, has limited opportunities for publics and policy-makers to engage with the affective landscapes of animal research and the humans and animals who inhabit them. An atmosphere of secrecy and security, developed as a consequence of attacks on animal researchers and suppliers in the 1980s and 1990s, endures in the imagination of both researchers and the public, and has been embedded in UK legislation through the so-called secrecy clause of ASPA.94 There are now moves towards greater openness, in part prompted by the perceived decline in public trust observed in MORI polls in the early 2000s. The Concordat on Openness encourages those working in animal research to commit to being ‘more open about the ways in which animals are used in scientific, medical and veterinary research in the UK’.95 This political move both recognises the apparent amplification of public fear that comes with secrecy and seeks to shift the burden of public scrutiny from those who are open about their work, to those who choose to remain guarded. The concordat was thus a deliberate attempt to shift the political atmospheres around animal research, and has now become the focus for further debate around the emotional honesty of how to account for the harms and benefits of animal research. This move to openness also creates opportunities for conversations both between stakeholders in animal research and with wider publics, and the chance to attend more carefully to how trust and other affects flow around the wider animal research nexus.

The changing nature, or lability, of affect also makes it a key domain in which to understand shifts across the animal research nexus. The histories and geographies of hope that have underpinned changing expectations for both scientific development and animal welfare improvements,96 the moves from atmospheres of secrecy towards greater openness,97 the increasing emphasis on cultures of care alongside the growing cultures of anxiety around expertise are critical to shifting relations across the animal research nexus. For example, the practice of rehoming laboratory animals has grown in part as a way of cultivating hopefulness and ‘life after the laboratory’ for animals and for those working at the facility, which feeds into achieving, or enhancing, a culture of care. In addition to work focusing on the small-scale affective inter-relations between humans and animals in immediate ‘face-to-face’, or perhaps rather ‘body-to-body’, encounters in the laboratory, we suggest research needs to explore the affective relations of animal research at a range of scales and temporalities: from the laboratory to the institution, the nation, the international context and over time. What happens, for example, if we broaden our concern with societal sentience98 to incorporate an attention to societal sensing, or the ways in which affect shapes engagements with animal research beyond the confines of the animal research facility? Currently, we might speculate that such sensings are dominated by the highly emotive campaign materials used in animal rights activism. Such materials (and the often hostile atmospheres they evoke) can be highly effective in attracting public scrutiny of animal research, but they can also close down conversations and limit the possibilities for policy-makers, patients and publics to engage with the complex, challenging and contradictory emotional landscapes of animal research, which do involve pain, suffering and harm, but also care, love and curiosity.

We are seeking such openings in our own work, through the development of innovative public engagement techniques, which support more comfortable conversations,99 as well as more challenging immersive experiences100 to open up the affective experiences of animal research to wider conversations. In short, nexus thinking, and experimenting with how to perform nexus relations,101 allows us to engage critically and constructively with both animal research and wider public sensibilities.

## Conclusions

In this paper, we have sought to introduce the concept of the animal research nexus as a new way of characterising complex relations around animal research. We demonstrated how nexus can function as an analytical device for mapping how specific inter-relations between scientific research, animal welfare and health: (i) have come into being through historical contingencies and path dependencies; (ii) are managed through formal and informal mechanisms of governance and (iii) shift through the affective dimensions of animal research. At each point, we have sought to navigate a way between recognition of the entangled relations and polarised public debates around animal research, by tracing the patterns of inclusion and exclusion around the UK animal research nexus. The account we have offered does not seek to supersede earlier work on animal research in the social sciences and humanities, but to provide a way of bringing this work into further conversations with policy and comparatively. Our analysis does likely differ from others in providing a stronger role for the policy processes and practices of governance in which UK research is embedded. In so doing, we have sought to further our commitments to supporting dialogue between academic research and policy communities and span the gap between critical academic analysis and collaborative research with stakeholders. A nexus approach, we argue, enables knowledge to travel between disciplines, policy and the public, and it reminds us that the specific nexus between science, health and animal welfare in the UK is situated and contingent, creating space for comparative analysis too. In conclusion, we reflect on the value of nexus concepts as a methodology for working within the Animal Research Nexus Programme, and potentially beyond.

First, we would highlight the value of a nexus approach for facilitating collaboration within our interdisciplinary research programme. The animal research nexus puts a central proposition about the relationality of animal research at the heart of our work and demands we communicate and collaborate across projects, institutions and disciplines in deep, sustained and transformative ways. The nexus becomes ‘a shifting place of dialogue, debate and reconfiguration’102 that enables projects to develop in conversation with, but not reducible to, each other.

Second, we propose the value of nexus approaches for facilitating interactive collaboration with wider stakeholders, including regulators, biomedical researchers, laboratory animal science and animal protection groups. There are two main aspects to this. One is the potential to identify and address questions that currently fall between disciplines. This might include understanding the continued dislocation between biomedical science and animal welfare science, with their distinct disciplinary histories, or the challenge of creating a space for the full consideration of alternatives to animal research within scientific communities and infrastructures that have been organised around animal models since the 1980s. Second is the ability to give space to the social relations, which stakeholders recognise as both central to animal research and difficult to engage with. Work on the animal research nexus cannot resolve these challenges, but it can identify the key parameters to them and help develop the relationships and methods that engage new voices and evolve the conversations.

Third, we want to acknowledge that there are challenges with applying a nexus approach that demand reflexivity. The pro-position and anti-position that we are seeking to move beyond are part of the historical inheritances of researching animal research. Many researchers and others involved in animal research are still cautious about having conversations across polarised positions and some of the wider publics and patients that we are engaging with are sceptical about moving beyond them. While seeking to bring new networks together, feelings of unease may be magnified. Many roles in this wider landscape of research are defined in relation to this history: for example, the patient or public involvement practitioner at a research institution looking to expand conversations around animal research may find they are blocked by their press office and concern about public relations. Understanding these historical investments in roles and relations is not an obstacle to a nexus approach overall, but a prompt to working reflexively to understand them and explore when and how it is appropriate to address them.

Finally, in working in an interdisciplinary, interactive and reflexive way with nexus approaches, we contend our programme has the potential to inform the application of this framework to other areas where there are complex intersections between science, society and governance. Particularly, we suggest that the medical humanities have the potential to enrich the dialogues around nexus thinking at the science/society interface. We have outlined here that the potential of a nexus approach can be deepened through recognising path dependencies and historical trajectories, and attending carefully to the operation and distribution of affect as key dynamics in understanding how a nexus may change. We have demonstrated that in animal research, and elsewhere, an approach to issues that cross human-animal or human-environment domains cannot be managed by science alone. We have indicated that nexus approaches can help understand how complexities are managed, who and what are included and excluded and how this may change over time. A nexus approach cannot resolve the questions this then raises, for the debate around animal research is not going to end. Underpinning our argument is a commitment to forms of analysis and critique, in animal research and elsewhere, that render these complex connections legible and to support the institutions that govern them to act in ways that are ultimately more responsive and accountable to this complexity.

## Notes

1. Wellcome Trust, ‘Strategic Plan 2010-2020: Extraordinary Opportunities’ (London: Wellcome Trust, n.d.), 17, https://dlcs.io/file/wellcome/1/2165ff54-cd4e-4e8d-a260-a393240447fe.

2. Robert G. W. Kirk (2010), ‘A Brave New Animal for a Brave New World: The British Laboratory Animals Bureau and the Constitution of International Standards of Laboratory Animal Production and Use, circa 1947–1968’, *Isis* 101, no. 1 (2010): 62–94.

3. Donna J Haraway (2008), *When Species Meet*, 3 (Minneapolis: University of Minnesota Press, 2008).

4. Beth Greenhough and Emma Roe (2019), ‘Exploring the Role of Animal Technologists in Implementing the 3Rs: An Ethnographic Investigation of the UK University Sector’, *Science, Technology, & Human Values* 43, no. 4 (2018): 694–722.

5. Linda Birke, Arnold Arluke, and Mike Michael (2007), *The Sacrifice: How Scientific Experiments Transform Animals and People* (Lafayette: Purdue University Press, 2007).

6. Pru Hobson-West and Ashley Davies (2018), ‘Societal Sentience: Constructions of the Public in Animal Research Policy and Practice’, *Science, Technology, & Human Values* 43, no. 4 (2018): 671–93; Carrie Friese (2013b), ‘Realizing Potential in Translational Medicine: The Uncanny Emergence of Care as Science’, *Current Anthropology* 54, no. S7 (2013): S129–38.

7. Sarah J. Whatmore (2009), ‘Mapping Knowledge Controversies: Science, Democracy and the Redistribution of Expertise’, *Progress in Human Geography* 33, no. 5 (2009): 587–98.

8. Andrew Barry, Georgina Born, and Gisa Weszkalnys (2008), ‘Logics of Interdisciplinarity’, *Economy and Society* 37, no. 1 (2008): 20–49.

9. Andrew Barry, Georgina Born, and Gisa Weszkalnys (2008), Born, and Weszkalnys, 40.

10. Felix Dodds and Jamie Bartram (2016), *The Water, Food, Energy and Climate Nexus: Challenges and an Agenda for Action* (Routledge, 2016).

11. Francesca Artioli, Michele Acuto, and Jenny McArthur (2017), ‘The Water-Energy-Food Nexus: An Integration Agenda and Implications for Urban Governance’, *Political Geography* 61 (2017): 215–23; Rose Cairns and Anna Krzywoszynska (2016), ‘Anatomy of a Buzzword: The Emergence of “the Water-Energy-Food Nexus” in UK Natural Resource Debates’, *Environmental Science & Policy* 64, no. Supplement C (2016): 164–70.

12. Francesca Artioli, Michele Acuto, and Jenny McArthur (2017), ‘The Water-Energy-Food Nexus’, 215.

13. Joe Williams, Stefan Bouzarovski, and Erik Swyngedouw (2014), ‘Politicising the Nexus: Nexus Technologies, Urban Circulation and the Coproduction of Water–Energy’, *Nexus Network Think Piece Series, Paper* 1 (2014): 5.

14. Felix Dodds and Jamie Bartram (2016), *The Water, Food, Energy and Climate Nexus*.

15. Francesca Artioli, Michele Acuto, and Jenny McArthur (2017), ‘The Water-Energy-Food Nexus’; Cairns and Krzywoszynska, ‘Anatomy of a Buzzword’.

16. Joe Williams, Stefan Bouzarovski, and Erik Swyngedouw (2014), ‘Politicising the Nexus’.

17. Christian Stein, Jennie Barron, and Timothy Moss (2014), Governance of the Nexus: From Buzz Words to a Strategic Action Perspective’, *Nexus Network Think Piece Series, Paper* 3 (2014).

18. Joe Williams, Stefan Bouzarovski, and Erik Swyngedouw (2014), ‘Politicising the Nexus’, 4.

19. Christian Stein, Jennie Barron, and Timothy Moss (2014), ‘Governance of the Nexus’, 8.

20. Andrew Stirling (2015), ‘Developing “Nexus Capabilities”: Towards Transdisciplinary Methodologies’, Reports and working papers, 30 June 2015, http://www.thenexusnetwork.org/new-discussion-paper-on-transdisciplinary-nexus-methods-from-andy-stirling/.

21. Rose Cairns and Anna Krzywoszynska (2016), ‘Anatomy of a Buzzword’; Dennis Wichelns (2017), ‘The Water-Energy-Food Nexus: Is the Increasing Attention Warranted, from Either a Research or Policy Perspective?’, *Environmental Science & Policy* 69 (2017): 113–23; Nature (2016), ‘Scientific Buzzwords Obscure Meaning’, *Nature News* 538, no. 7624 (2016): 140.

22. Tafadzwanashe Mabhaudhi, Tendai Chibarabada, and Albert Modi (2016), ‘Water-Food-Nutrition-Health Nexus: Linking Water to Improving Food, Nutrition and Health in Sub-Saharan Africa’, *International Journal of Environmental Research and Public Health* 13, no. 1 (2016): 107; William A Masters et al. (2014), ‘Agriculture, Nutrition, and Health in Global Development: Typology and Metrics for Integrated Interventions and Research’, *Annals of the New York Academy of Sciences* 1331, no. 1 (2014): 258–69.

23. Waithaka Mwangi, Paul de Figueiredo, and Michael F. Criscitiello (2016), ‘One Health: Addressing Global Challenges at the Nexus of Human, Animal, and Environmental Health’, *PLOS Pathogens* 12, no. 9 (2016).

24. Peter Jackson (2016), ‘New Directions in Cultural Geography Revisited’, *Area* 48, no. 3 (2016): 369.

25. Carrie Friese (2019), ‘Intimate Entanglements in the Animal House: Caring for and about Mice’, *The Sociological Review* 67, no. 2 (2019): 287–98; Carrie Friese, Nathalie Nuyts, and ‘Posthumanist Critique (2017), ‘Posthumanist Critique and Human Health: How Nonhumans (Could) Figure in Public Health Research’, *Critical Public Health* 27, no. 3 (2017): 303–13.

26. Joe Williams, Stefan Bouzarovski, and Erik Swyngedouw (2014), ‘Politicising the Nexus’, 19.

27. Christian Stein, Jennie Barron, and Timothy Moss (2014), ‘Governance of the Nexus’, 6.

28. Peter Jackson (2016), ‘New Directions in Cultural Geography Revisited’, 369.

29. Joe Williams, Stefan Bouzarovski, and Erik Swyngedouw (2014), ‘Politicising the Nexus’, 20.

30. Rachel Blau DuPlessis and Peter Quartermain (1999) Objectivist Nexus: Essays in Cultural Poetics (Tuscaloosa: University of Alabama Press, 1999).

31. Rachel Blau DuPlessis and Peter Quartermain (1999), 22.

32. Rachel Blau DuPlessis and Peter Quartermain (1999), 22.

33. Tim Schwanen (2018), ‘Thinking Complex Interconnections: Transition, Nexus and Geography’, *Transactions of the Institute of British Geographers* 43, no. 2 (2018): 262–83.

34. Rachel Blau DuPlessis and Peter Quartermain (1999), *The Objectivist Nexus*, 17.

35. Michael E. Lynch (1988), ‘Sacrifice and the Transformation of the Animal Body into a Scientific Object: Laboratory Culture and Ritual Practice in the Neurosciences’, *Social Studies of Science* 18 (1988): 265–289.

36. Beth Greenhough and Emma Roe (2019), ‘Exploring the Role of Animal Technologists in Implementing the 3Rs’; Linda Birke, Arnold Arluke, and Mike Michael (2007), *The Sacrifice: How Scientific Experiments Transform Animals and People*; Tone Druglitrø (2018), ‘“Skilled Care” and the Making of Good Science’, *Science, Technology, & Human Values* 43, no. 3 (2018): 649–70; Eva Giraud and Gregory Hollin (2016), ‘Care, Laboratory Beagles and Affective Utopia’, *Theory, Culture & Society* 33, no. 4 (2016): 27–49; Mette N. Svendsen and Lene Koch (2013), ‘Potentializing the Research Piglet in Experimental Neonatal Research’, *Current Anthropology* 54, no. S7 (2013): S118–28 Rachel A Ankeny et al. (2014), ‘Making Organisms Model Human Behavior: Situated Models in North-American Alcohol Research, since 1950’, *Science in Context* 27, no. 3 (2014): 485–509; Tora Holmberg and Tora Holmberg (2011), ‘Mortal Love: Care Practices in Animal Experimentation’, ed. Myra J. Hird, *Feminist Theory* 12, no. 2 (2011): 147–63; Gail Davies (2013a), ‘Mobilizing Experimental Life: Spaces of Becoming with Mutant Mice’, *Theory, Culture & Society* 30, no. 7–8 (2013): 129–53; Gail Davies (2013), ‘Arguably Big Biology: Sociology, Spatiality and the Knockout Mouse Project’, *BioSocieties* 8, no. 4 (2013): 417–31.

37. Tone Druglitrø and Robert G W Kirk (2014), ‘Building Transnational Bodies: Norway and the International Development of Laboratory Animal Science, ca. 1956–1980’, *Science in Context* 27, no. 2 (2014): 333–57.

38. Lynda Birke (2012), ‘Animal Bodies in the Production of Scientific Knowledge: Modelling Medicine’, *Body & Society* 18 (2012): 156–178.

39. Mie S. Dam and Mette N. Svendsen (2018), ‘Treating Pigs: Balancing Standardisation and Individual Treatments in Translational Neonatology Research’, *BioSocieties* 13, no. 2 (2018): 349–67.

40. Rachel A Ankeny (2010), ‘Historiographic Reflections on Model Organisms: Or How the Mureaucracy May Be Limiting Our Understanding of Contemporary Genetics and Genomics’, *History and Philosophy of the Life Sciences*, 2010, 91–104.

41. Robert E Kohler (1994), *Lords of the Fly: Drosophila Genetics and the Experimental Life* (Chicago: University of Chicago Press, 1994); Robert G. W. Kirk (2010), ‘A Brave New Animal for a Brave New World’; Rachel A. Ankeny and Sabina Leonelli (2011), ‘What’s so Special about Model Organisms?’, *Studies in History and Philosophy of Science Part A*, Model-Based Representation in Scientific Practice, 42, no. 2 (2011): 313–23; Christopher M Kelty (2012), ‘This Is Not an Article: Model Organism Newsletters and the Question of “Open Science”’, *BioSocieties* 7 (2012): 140–168.

42. Dan Lyons (2011), ‘Protecting Animals versus the Pursuit of Knowledge: The Evolution of the British Animal Research Policy Process’, *Society & Animals* 19, no. 4 (2011): 356–67.

43. Rhoda Wilkie (2015), ‘Multispecies Scholarship and Encounters: Changing Assumptions at the Human-Animal Nexus’, *Sociology* 49, no. 2 (2015): 323–39.

44. Jessie L. Krienert et al. (2012), ‘Examining the Nexus Between Domestic Violence and Animal Abuse in a National Sample of Service Providers’, *Violence and Victims; New York* 27, no. 2 (2012): 280–95.

45. Melanie J. Rock (2017), ‘Who or What Is “the Public” in Critical Public Health? Reflections on Posthumanism and Anthropological Engagements with One Health’, *Critical Public Health* 27, no. 3 (2017): 314–24; Victor Galaz et al. (2015), *The Political Economy of One Health Research and Policy* (STEPS Centre, 2015), https://opendocs.ids.ac.uk/opendocs/handle/123456789/6598.

46. J A Blake et al. (2001), ‘The Mouse Genome Database (MGD): Integration Nexus for the Laboratory Mouse’, *Nucleic Acids Research* 29, no. 1 (2001): 91–94.

47. Vonne Lund et al. (2006), ‘Animal Welfare Science—Working at the Interface between the Natural and Social Sciences’, *Applied Animal Behaviour Science*, International Society for Applied Ethology (ISAE) Special Issue 2004, 97, no. 1 (2006): 37–49.

48. Wesley V. Jamison and William M. Lunch (1992), ‘Rights of Animals, Perceptions of Science, and Political Activism: Profile of American Animal Rights Activists’, *Science, Technology, & Human Values* 17, no. 4 (1992): 441.

49. Mike Michael and Lynda Birke (1994), ‘Enrolling the Core Set: The Case of the Animal Experimentation Controversy’, *Social Studies of Science* 24, no. 1 (1994): 85–86.

50. Nature (2016), ‘Scientific Buzzwords Obscure Meaning’.

51. Elizabeth R Johnson (2015), ‘Of Lobsters, Laboratories, and War: Animal Studies and the Temporality of More-Than-Human Encounters’, *Environment and Planning D: Society and Space* 33, no. 2 (2015): 296–313.

52. Donna J Haraway (2008), *When Species Meet*.

53. Gail Davies et al. (2018), ‘Science, Culture, and Care in Laboratory Animal Research: Interdisciplinary Perspectives on the History and Future of the 3Rs’, *Science, Technology, & Human Values* 43, no. 4 (2018): 603–21.

54. Carmen McLeod and Pru Hobson-West (2016), ‘Opening up Animal Research and Science–Society Relations? A Thematic Analysis of Transparency Discourses in the United Kingdom’, *Public Understanding of Science* 25, no. 7 (2016): 791–806.

55. Jack Stilgoe and James Wilsdon (2008), ‘The New Politics of Public Engagement with Science?’, in *Investigating Science Communication in the Information Age: Implications for Public Engagement and Popular Media*, ed. Richard Holliman et al. (Oxford: Oxford University Press, 2008).

56. Gail Davies (2012), ‘What Is a Humanized Mouse? Remaking the Species and Spaces of Translational Medicine’, *Body & Society* 18, no. 3–4 (2012): 126–55.

57. Richard Horton (2015), ‘Offline: What Is Medicine’s 5 Sigma?’, *The Lancet* 385, no. 9976 (2015): 1380.

58. Richard Jones and James Wilsdon (2018), ‘The Biomedical Bubble’, nesta, 2018, https://www.nesta.org.uk/report/biomedical-bubble/.

59. Gail F Davies et al. (2016), ‘Developing a Collaborative Agenda for Humanities and Social Scientific Research on Laboratory Animal Science and Welfare’, *PLOS ONE* 11, no. 7 (2016).

60. Robert G. W Kirk (2018), ‘Recovering The Principles of Humane Experimental Technique: The 3Rs and the Human Essence of Animal Research’, *Science, Technology, & Human Values* 43, no. 4 (2018): 622–48.

61. P Hobson-West (2010), ‘The Role of “Public Opinion” in the UK Animal Research Debate’, *Journal of Medical Ethics* 36, no. 1 (2010): 46–49.

62. Robert E Kohler (1994), *Lords of the Fly*; Ankeny and Leonelli, ‘What’s so Special about Model Organisms?’; Karen A Rader (2004), *Making Mice: Standardizing Animals for American Biomedical Research, 1900-1955* (Princeton: Princeton University Press, 2004); Angela Cassidy et al. (2017), ‘Animal Roles and Traces in the History of Medicine, c.1880–1980’, *BJHS Themes* 2 (2017): 11–33 Abigail Woods et al. (2018), ‘Annotated Bibliography of Animals in the History of Medicine’, in *Animals and the Shaping of Modern Medicine* (Cham: Springer, 2018), 247–268.

63. Robert G. W. Kirk (2010), ‘A Brave New Animal for a Brave New World’; Kirk, ‘Recovering The Principles of Humane Experimental Technique’; Robert G.W. Kirk (2008), ‘“Wanted-Standard Guinea Pigs”: Standardisation and the Experimental Animal Market in Britain ca. 1919-1947’, *Studies in History and Philosophy of Biological and Biomedical Sciences* 39 (2008): 280–291.

64. Erica Fudge (2002), ‘A Left-Handed Blow: Writing the History of Animals’, in *Representing Animals*, ed. Nigel Rothfels (Bloomington: Indiana University Press, 2002).

65. Chris Pearson (2015), ‘Beyond “Resistance”: Rethinking Nonhuman Agency for a “More-than-Human” World’, *European Review of History: Revue Européenne d’histoire* 22, no. 5 (2015): 709–25.

66. Susan Nance (2013), *Entertaining Elephants: Animal Agency and the Business of the American Circus* (Baltimore: The Johns Hopkins University Press, 2013); Abraham Gibson (2016), *Feral Animals in the American South: An Evolutionary History* (New York, NY: Cambridge University Press, 2016); Erica Fudge (2017), ‘What Was It Like to Be a Cow? History and Animal Studies’, in *The Oxford Handbook of Animal Studies*, ed. Linda Kalof (New York: Oxford University Press, 2017).

67. Gregory Hollin et al. (2017), ‘(Dis)Entangling Barad: Materialisms and Ethics’, *Social Studies of Science* 47, no. 6 (2017): 918–41.

68. Pru Hobson-West (2012), ‘Ethical Boundary-Work in the Animal Research Laboratory’, *Sociology* 46, no. 4 (2012): 649–63.

69. Dan Lyons (2011), ‘Protecting Animals versus the Pursuit of Knowledge’.

70. Dan Lyons (2011), 357.

71. S Raman et al. (2018) et al., ‘'Science Matters’ and the Public Interest: The Role of Minority Engagement’, in *Science and the Politics of Openness*, ed. B Nerlich et al. (Manchester: Manchester University Press, 2018).

72. Pru Hobson-West and Ashley Davies (2018), ‘Societal Sentience’.

73. John Webster (2014), ‘Ethical and Animal Welfare Considerations in Relation to Species Selection for Animal Experimentation’, *Animals* 4, no. 4 (2014): 729–41.

74. Animals (Scientific Procedures) Act (1986), section 5b(d), https://www.legislation.gov.uk/ukpga/1986/14/contents.

75. Bernard Foëx (2007), ‘The Ethics of Animal Experimentation’, *Emergency Medicine Journal* 24, no. 11 (2007): 750–51.

76. Gail F Davies (2018), ‘Harm-Benefit Analysis: Opportunities for Enhancing Ethical Review in Animal Research’, *Lab Animal* 47, no. 3 (2018): 57.

77. Gregory Hollin et al. (2017), ‘(Dis)Entangling Barad’, 932.

78. V Ashall and P. Hobson-West (2018), ‘The Vet in the Lab: Exploring the Position of Animal Professionals in Non-Therapeutic Roles’, in *Professionals in Food Chains*, ed. S Springer and Herwig Grimm (The Netherlands: Wageningen Academic Publishers, 2018), 291–95.

79. P Hobson-West (2010), ‘The Role of “Public Opinion” in the UK Animal Research Debate’.

80. G. F Davies, R. Gorman, and B. Crudgington (2019), ‘Which Patient Takes Centre Stage? Placing Patient Voices in Animal Research’, in *GeoHumanities and Health*, ed. Sarah Atkinson and R Hunt (Springer, 2019).

81. Gail F Davies (2018), ‘Harm-Benefit Analysis’.

82. Jamie Lorimer, Timothy Hodgetts, and Maan Barua (2019), ‘Animals’ Atmospheres’, *Progress in Human Geography* 43, no. 1 (2019): 26–45; Ben Anderson (2009), ‘Affective Atmospheres’, *Emotion, Space and Society* 2, no. 2 (2009): 77–81.

83. Katrina Brown et al. (2019), ‘Empathy, Place and Identity Interactions for Sustainability’, *Global Environmental Change* 56 (2019): 11–17.

84. Carrie Friese (2019), ‘Intimate Entanglements in the Animal House’; Eva Hayward (2010), ‘FINGERYEYES: Impressions of Cup Corals’, *Cultural Anthropology* 25, no. 4 (2010): 577–99.

85. Donna J Haraway (2008), *When Species Meet*.

86. Vinciane Despret (2004), ‘The Body We Care for: Figures of Anthropo-Zoo-Genesis’, *Body & Society* 10, no. 2–3 (2004): 111–34; Vinciane Despret (2013), ‘Responding Bodies and Partial Affinities in Human–Animal Worlds’, *Theory, Culture & Society* 30, no. 7–8 (2013): 51–76.

87. Robert G. W Kirk (2014), ‘The Invention of the “Stressed Animal” and the Development of a Science of Animal Welfare, 1947–86’, in *Stress, Shock, and Adaptation in the Twentieth Century*., ed. D Cantor and Edmund Ramsden (Rochester (NY): University of Rochester Press, 2014), 241–63.

88. Mara Miele (2016), ‘The Making of the Brave Sheep or … the Laboratory as the Unlikely Space of Attunement to Animal Emotions’, *GeoHumanities* 2, no. 1 (2016): 58–75.

89. Beth Greenhough and Emma Roe (2018), ‘Exploring the Role of Animal Technologists in Implementing the 3Rs’; Beth Greenhough and Emma Roe (2011), ‘Ethics, Space, and Somatic Sensibilities: Comparing Relationships between Scientific Researchers and Their Human and Animal Experimental Subjects’, *Environment and Planning D: Society and Space* 29, no. 1 (2011): 47–66; Beth Greenhough and Emma Roe (2019), ‘Attuning to Laboratory Animals and Telling Stories: Learning Animal Geography Research Skills from Animal Technologists’, *Environment and Planning D: Society and Space* 37, no. 2 (2019): 367–84.

90. Bernard H. Gustin (1973), ‘Charisma, Recognition and the Motivation of Scientists’, *American Journal of Sociology* 78, no. 5 (1973): 1119–34; Robert Merton (1973), *The Sociology of Science: Theoretical and Empirical Investigations* (Chicago, IL: University of Chicago Press, 1973).

91. Liz Bondi (2005), ‘The Place of Emotions in Research’, in *Emotional Geographies* (Hampshire: Ashgate Publishing, 2005), 231–46; Richard Phillips (2010), ‘The Impact Agenda and Geographies of Curiosity’, *Transactions of the Institute of British Geographers* 35, no. 4 (2010): 447–52.

92. Lorraine Daston (1995), ‘The Moral Economy of Science’, *Osiris* 10 (1995): 4.

93. G. F Davies, R. Gorman, and B. Crudgington (2019), ‘Which Patient Takes Centre Stage?’

94. Pandora Pound and Ricardo Blaug (2016), ‘Transparency and Public Involvement in Animal Research’, *Alternatives to Laboratory Animals: ATLA* 44, no. 2 (2016): 167–73.

95. Understanding Animal Research (2017), ‘Concordat on Openness on Animal Research in the UK’, Concordat on Openness on Animal Research in the UK, 2017, 4, http://concordatopenness.org.uk/wp-content/uploads/2017/04/Concordat-Final-Digital.pdf.

96. Nuno Henrique Franco, Peter Sandøe, and I. Anna S. Olsson (2018), ‘Researchers’ Attitudes to the 3Rs—An Upturned Hierarchy?’, *PLoS ONE* 13, no. 8 (2018).

97. Graham Henry (2012), ‘A Unique Look inside Cardiff University’s Secretive Animal-Testing Centre’, walesonline, 31 October 2012, http://www.walesonline.co.uk/news/local-news/unique-look-inside-cardiff-universitys-2023216.

98. Pru Hobson-West and Ashley Davies (2018), ‘Societal Sentience’.

99. Richard Gorman and Gail Davies (2019), ‘Patient and Public Involvement and Engagement (PPIE) with Animal Research’, accessed 28 August 2019, https://animalresearchnexus.org/publications/patient-and-public-involvement-and-engagement-ppie-animal-research; Animal Research Annex (2019), ‘Mouse Exchange @ Southampton Science and Engineering Festival’, accessed 28 August 2019, https://animalresearchnexus.org/events/mouse-exchange-southampton-science-and-engineering-festival.

100. Vector (2019), Animal Research Nexus’, accessed 10 June 2019, https://animalresearchnexus.org/events/vector.

101. Lauren Berlant and Kathleen Stewart (2019), For one possible mode of nexus writing that links affect, politics, and attention to processes of world-making see Berlant and StewartBerlant, *The Hundreds:* (Durham: Duke University Press, 2019).

102. Rachel Blau DuPlessis and Peter Quartermain (1999) and Quartermain, *The Objectivist Nexus*, 22.

103. M Brown (2014), ‘Creating a Culture of Care’, NC3Rs News & Blog Online., 2014, https://www.nc3rs.org.uk/news/creating-culture-care; RSPCA and LASA (2015), ‘Guiding Principles on Good Practice for Animal Welfare and Ethical Review Bodies’, 2015, http://www.lasa.co.uk/PDF/AWERB_Guiding_Principles_2015_final.pdf; Marilyn J Brown et al. (2018), ‘Culture of Care: Organizational Responsibilities’, in *Management of Animal Care and Use Programs in Research, Education, and Testing*, ed. Robert H. Weichbrod, Gail A. (Heidbrink) Thompson, and John N. Norton, 2nd ed. (Boca Raton (FL): CRC Press/Taylor & Francis, 2018).

104. Dawn Goodwin (2018), ‘Cultures of Caring: Healthcare “Scandals”, Inquiries, and the Remaking of Accountabilities’, *Social Studies of Science* 48, no. 1 (2018): 101–124.

105. Gail Davies et al. (2018) et al., ‘Science, Culture, and Care in Laboratory Animal Research’.

106. Ben Anderson (2009), ‘Affective Atmospheres’; Derek P. McCormack (2008), ‘Engineering Affective Atmospheres on the Moving Geographies of the 1897 Andrée Expedition’, *Cultural Geographies* 15, no. 4 (2008): 413–30.
